# Investigation of the Relevance of CYP3A4 Inhibition on the Pharmacokinetics of the Novel P2X3 Antagonist Filapixant: Results of In Vitro Explorations and a Fixed-Sequence Clinical Trial with Itraconazole in Healthy Volunteers

**DOI:** 10.3390/ijms262010177

**Published:** 2025-10-20

**Authors:** Klaus Francke, Antje Rottmann, Stefan Klein, Joachim Höchel, Christian Friedrich

**Affiliations:** 1 Research and Development, Pharmaceuticals, Bayer AG, 13353 Berlin, Germanyantje_metab.rottmann@bayer.com (A.R.); joachim.hoechel@bayer.com (J.H.); 2 MHB-Medizinische Hochschule Brandenburg, 14770 Brandenburg, Germany

**Keywords:** drug–drug interaction, filapixant, itraconazole, pharmacokinetic, P2X3-anatgonist

## Abstract

Hypersensitized P2X3 receptor signaling has been described to play a role in several disorders, including chronic cough. The goal of our in vitro and in vivo studies was to investigate the biotransformation and the influence of CYP3A4 inhibition on the pharmacokinetics of the selective P2X3 antagonist filapixant. Metabolic turnover of filapixant in human liver microsomes and hepatocytes was moderate to high, indicating a complex metabolic pattern with mainly oxidative biotransformation. In recombinant CYP enzymes, depletion of filapixant was observed mainly with CYP3A4 and, to a significantly lesser extent, with CYP1A1, 2D6, 2J2, and 3A5. Drug depletion of [^3^H]filapixant and metabolite formation in human liver microsomes was significantly inhibited in the presence of strong CYP3A4 inhibitors, whereas other CYP isoform–selective inhibitors showed no or very minor effects. Co-administration of multiple daily doses of 200 mg itraconazole with 80 mg filapixant in humans increased the AUC and C_max_ of filapixant to 4.01 and 1.89-fold, respectively, indicating that filapixant is a moderately sensitive CYP3A4 substrate. Co-administration of itraconazole also prolonged the half-life of filapixant from 12.1 h to 22.8 h. Overall, changes in AUC, C_max_, and half-life indicate that both the bioavailability and elimination of filapixant were affected. Filapixant was well tolerated alone and in combination with itraconazole.

## 1. Introduction

Several P2X3 receptor antagonists have been in development for the management of refractory chronic cough and have also been tested for other conditions such as endometriosis, osteoarthritis, and bladder disorders. The first clinically investigated P2X3 antagonist, gefapixant, reported efficacy in chronic cough, osteoarthritis, and bladder pain syndrome, which was hampered by a high frequency of taste disturbances. These side effects were considered likely related to the blockade of the P2X2/3 receptor heteromer in the taste-sensing system. Therefore, P2X3-selective receptor antagonists have been developed with the hope to avoid those findings. This has been only partially achieved—the frequency of taste-related side effects with the selective P2X3 antagonist eliapixant was reported to be considerably reduced compared to gefapixant; however, taste disturbances were still seen in a relevant number of patients [1]. Interestingly, another newly developed and even more selective P2X3 antagonist, filapixant (BAY 1902607), showed a taste disturbance frequency being substantially higher than for eliapixant, indicating that other factors besides selectivity such as pharmacokinetic (PK) properties might play a role. Filapixant is a P2X3 antagonist with approximately 100-fold selectivity over the P2X2/3 heteromeric receptor developed for the indication of refractory chronic cough. It has been characterized in initial phase I and phase IIa studies, where it was overall safe and well tolerated and showed its potential in treating patients with refractory chronic cough [2,3]. In phase I studies, filapixant was rapidly absorbed, with maximum concentrations reached about 1–2 h after administration and showed dose-proportional and time-linear PK. The observed terminal half-life of ~6–10 h makes it suitable for once-daily treatment. Co-administration of filapixant had no relevant effect on the PK of midazolam in humans [3].

In this paper, we report the results of in vitro investigations on the metabolism of filapixant and the results of a subsequent clinical drug–drug interaction study with filapixant and itraconazole, a potent cytochrome P450 3A4 (CYP3A4) and P-glycoprotein (P-gp) inhibitor. The human study evaluated the drug–drug interaction potential with the strong CYP3A4/P-gp inhibitor itraconazole to allow dosing recommendations for comedications of filapixant in future clinical studies. Itraconazole was chosen as prototypical inhibitor according to relevant guidelines [4,5].

## 2. Results

### 2.1. In Vitro Metabolite Profiling and Enzymes Involved in Metabolism of Filapixant

**Figure 1:**
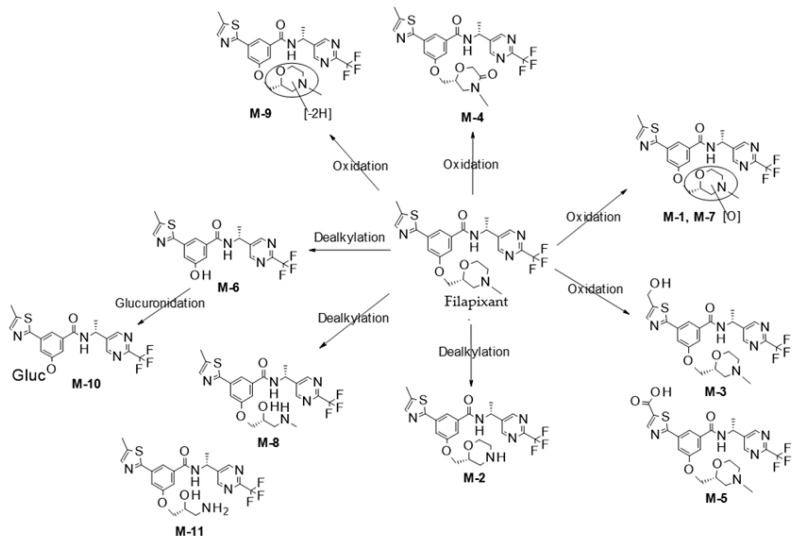
Proposed biotransformation pathways of filapixant.

The metabolic turnover of filapixant in human liver microsomes and hepatocytes was moderate to high showing a complex metabolic pattern (see Appendix C). Mainly oxidative biotransformation at the methyl morpholine moiety was observed (see Figure 1). Oxidation/hydroxylation forming M 1, M 4, and M 7 as well as dealkylation to M 2, M 6, and M 8 were the major primary metabolic pathways. Oxidation at the methyl thiazole moiety forming M 3 and subsequent oxidation to carboxylic acid M 5 was observed to a minor extent. In human hepatocytes, O dealkylation product M 6 is completely glucuronidated to M 10.

In recombinant cytochrome P450 (CYP) enzymes, relevant depletion of filapixant was observed, mainly applying CYP3A4 and to a substantially lesser extent also with CYP1A1, 2D6, 2J2, and 3A5 (Table 1). The apparent intrinsic clearance (CL_int_) of filapixant at concentrations of 0.1 and 1 μM was determined with CYP isoforms (1A1, 2D6, 2J2, and 3A4), thus confirming the dominant role of CYP3A4, with CL_int_ values at least 10-fold higher relative to the other CYP isoforms tested. Determination of CLint of filapixant in recombinant CYP1A1, CYP2D6, CYP2J2, and CYP3A4 resulted in the following CLint values: 0.217, 0.0618, and 0.126 and 1.32 µL/min/pmol CYP, respectively (for 1 µM). Incubation of 0.1 µM filapixant in the presence of CYP1A1, CYP2D6, and CYP2J2 resulted in comparable CLint values: 0.252, 0.0716, and 0.132 and 1.66 µL/min/pmol CYP, respectively.

Drug depletion of [^3^H]filapixant and the formation of metabolites in human liver microsomes was inhibited in the presence of strong CYP3A4 inhibitors (itraconazole, mibefradil, and azamulin), while other CYP isoform–selective inhibitors showed no or very minor inhibitory effects on filapixant biotransformation.

**Figure 2:**
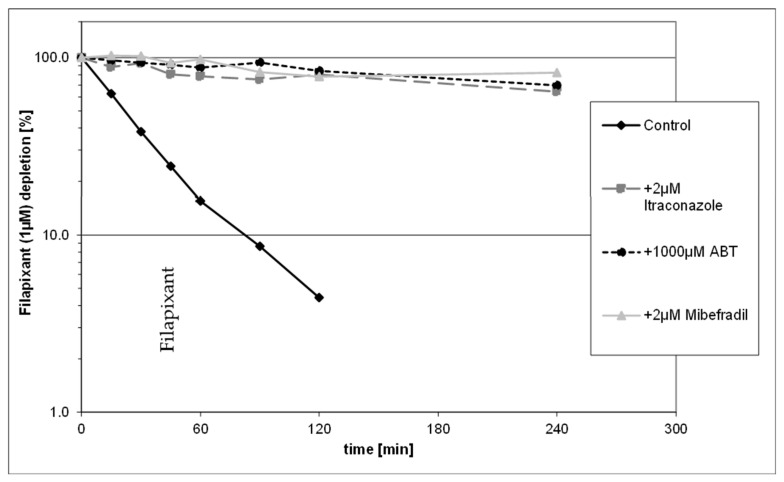
Depletion of 1 µM filapixant in human hepatocytes (1 × 10^6^ cells/mL) in the presence or absence of CYP3A4 selective inhibitors (itraconazole, mibefradil) and pan-CYP inhibitor (1-Aminobenzotriazol (ABT)).

Incubation of 1 µM filapixant in human hepatocytes in presence and absence of CYP isoform selective inhibitors underlined the results from previous investigations. In the presence of strong CYP3A4 inhibitors (mibefradil and itraconazole) as well as the pan-CYP inhibitor aminobenzotriazole (ABT), the apparent CL_int_ of filapixant was reduced by about 90 to 95% compared to untreated control, thus confirming the dominant role of CYP3A4 (Figure 2). The concentration of 2 µM itraconazole in the in vitro assay is in the range of the itraconazole plasma concentration in the clinical DDI study (see Section 2.2.1)

### 2.2. Clinical Study

**Figure A1:**
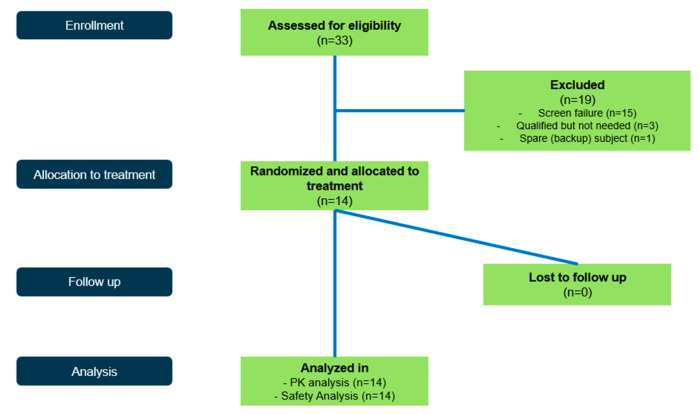
Participant disposition.

Overall, 33 participants were screened. Of these, 19 participants were screening failures, and 14 participants were assigned to treatment and completed the study (Figure A1).

Participants were white males with a mean age (±standard deviation, SD) of 47.1 ± 6.2 years, and a mean body mass index (±SD) of 24.5 ± 2.1 kg/m^2^.

#### 2.2.1. Pharmacokinetic Results

**Figure 3:**
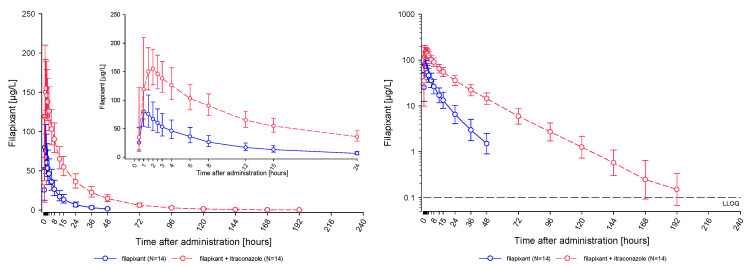
Geometric mean (and SD) concentration-time profiles of filapixant (μg/L) in plasma after single oral administration of 80 mg filapixant with and without concomitant administration of daily 200 mg itraconazole [linear scale left; semi-log scale right; N = 14; lower limit of quantification (LLOQ): 0.100 µg/L].

A single oral dose of 80 mg filapixant was rapidly absorbed under fasted conditions. Median time to reach maximum observed plasma concentrations of filapixant was approximately 1 h, without as well as with, itraconazole co-administration (Figure 3).

**Figure 4:**
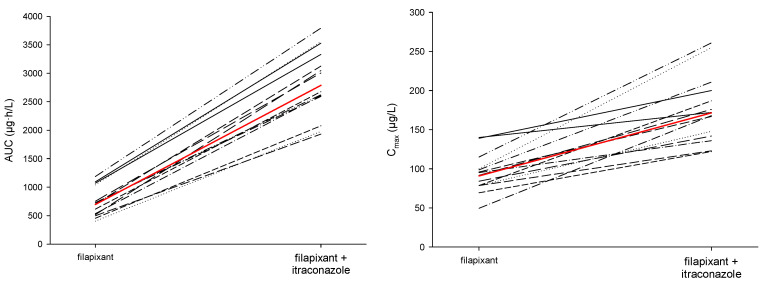
Stick plots of individual values of C_max_ and AUC of filapixant in plasma after single oral administration of 80 mg filapixant with and without concomitant administration of 200 mg itraconazole (red line connects Gmean data; dashed lines connect individual data points).

Co-administration of itraconazole markedly increased the systemic exposure of filapixant: The observed mean peak plasma concentrations (C_max_) were nearly twice as high after co-administration of itraconazole than after administration without itraconazole (172 µg/L and 90.8 µg/L, respectively). The observed mean exposure (AUC) was increased to a 4-times higher exposure (2790 µg·h/L vs. 695 µg·h/L; Table 2). As displayed in Figure 4, increases in exposure (AUC and C_max_) were consistent across participants.

Geometric mean terminal half-life (t_1/2_) of filapixant after single-dose administration was longer when filapixant was given together with itraconazole (22.8 h) compared to filapixant alone (12.1 h).

The point estimates (LS-means) and 2-sided 90% confidence intervals (CIs) of the main PK metrics AUC and C_max_ for filapixant resulting from an analysis of variance model are given in Table 3. Co-administration of the strong CYP3A4/P-gp inhibitor itraconazole markedly and significantly increased the systemic exposure of filapixant resulting in 1.89-fold higher mean C_max_ (90% CI: 1.67; 2.15) and 4.01-fold higher mean exposure (AUC, 90% CI: 3.66; 4.39).

Mean maximum concentrations of itraconazole were observed on study day 4 and were 1014.6 µg/L, indicating the achievement of adequate itraconazole exposure for CYP3A4/P-gp inhibition.

#### 2.2.2. Safety Results

In total, seven of the fourteen participants experienced treatment emergent adverse events (TEAEs). There was no apparent difference in the frequency, intensity, and character of the TEAEs between the two treatment periods of the study. The most frequently reported TEAE assessed as related to filapixant was headache (two participants). None of the participants reported any taste-related adverse event.

Overall, there were no clinically relevant trends in the safety laboratory parameters of the participating participants after the administration of filapixant with and without itraconazole. There were no clinically relevant findings observed in blood pressure, pulse rate measurements, and the electrocardiogram assessments.

## 3. Discussion

The overall aim of our investigations was to evaluate the drug–drug interaction potential of the strong CYP3A4/P-gp inhibitor itraconazole on the PK of orally administered filapixant to allow recommendations whether and how to use filapixant in the presence of CYP3A4 inhibitors in future studies and potentially in medical practice.

Incubations of 1 µM [^3^H]filapixant were performed in human liver microsomes and human hepatocytes in the presence or absence of CYP isoform–selective inhibitors. Drug depletion and formation of metabolites were inhibited in the presence of strong CYP3A4 inhibitors, while other CYP isoform–selective inhibitors showed no or very minor inhibitory effects on [^3^H]filapixant biotransformation. In the presence of strong CYP3A4 inhibitors, the apparent CL_int_ of filapixant was reduced by about 90 to 95% compared to untreated control. Thus, it was concluded that metabolization by CYP3A4 is the main elimination pathway for filapixant indicating a substantial risk for exposure increases in filapixant in the presence of CYP3A4 inhibitors in a clinical setting, and thus a clinical drug–drug interaction study was subsequently conducted.

Of note, filapixant shows in vitro a concentration dependent moderate to high permeability with a saturable efflux. This efflux is mediated by P-gp, thus filapixant is a P-gp substrate. Due to the saturable efflux at higher concentration and the moderate to high permeability, the overall effect of P-gp on the absorption of filapixant is considered to be of limited importance (Bayer AG; data on file).

For the clinical study and in accordance with relevant guidelines [4,5], itraconazole was chosen as a strong index CYP3A4/P-gp inhibitor to characterize a worst-case scenario. Itraconazole was given over 14 days with a single dose of filapixant co-administered one hour before the fourth itraconazole dose. Three days pretreatment were chosen to allow itraconazole to reach plasma concentrations leading to maximum CYP3A4 and P-gp inhibition before concomitant administration of filapixant. After the concomitant administration on day 4, itraconazole treatment was continued for another nine days to maintain maximum CYP3A4/P-gp inhibition during the whole elimination phase of filapixant, which was expected to be prolonged due to itraconazole related inhibition of the main metabolic pathway. The concomitant dosing of filapixant on day 4 was performed 1 h after itraconazole to guarantee maximum itraconazole concentrations and inhibition of CYP3A4 and P-gp at the gut wall during filapixant absorption.

The observed PK data demonstrated that a single oral dose of 80 mg filapixant was rapidly absorbed. The concomitant administration of itraconazole affected C_max_ of filapixant, but not t_max_. Median t_max_ of filapixant was about 1 h in both study periods. However, the range of individual t_max_ values was greater in the co-administration period, indicating an inter-individually variable effect of itraconazole on the absorption of filapixant.

Co-administration of the strong CYP3A4/P-gp inhibitor itraconazole markedly and significantly increased the systemic exposure of filapixant in all treated participants resulting in 1.89-fold mean peak concentrations (C_max_, 90% CI: 1.67; 2.15) and 4.01-fold mean exposure (AUC, 90% CI: 3.66; 4.39) compared to administration of filapixant alone. This effect confirmed the assumptions from prior in vitro studies with filapixant.

Geometric mean terminal half-life (t_1/2_) of filapixant after single-dose administration was—as expected—substantially longer in when participants received filapixant with itraconazole (22.8 h) than after administration of filapixant alone (12.1 h).

In summary, the changes in both C_max_ and half-life together with the overall change in AUC indicate that both clearance and bioavailability were affected by itraconazole and confirm predictions based on in vitro data. Considering the saturable efflux of filapixant due to P-gp dependent transport and the overall moderate to high permeability of the compound, the observed effect is primarily attributed to CYP3A4, while a minor contribution of P-gp cannot be excluded.

Observed maximum plasma concentrations of itraconazole on day 4 were similar to previously observed results and indicate adequate inhibition of CYP3A4 and P-gp [6,7].

Filapixant was well tolerated after a single oral dosing of 80 mg filapixant and in combination with multiple daily oral doses of 200 mg itraconazole. All TEAEs were of mild intensity. There was no apparent difference in the frequency of TEAEs with filapixant alone or combined with itraconazole, including taste-related adverse events. No relevant changes in vital signs, electrocardiograms, or laboratory values have been observed. The absence of taste-related adverse events is consistent with the previously reported multiple-dose escalation study, where taste-related adverse events were prominently seen with a dose leading to maximum concentrations above 400 µg/L, which is about 2-fold higher than the maximum concentrations reached when filapixant 80 mg was co-administered with itraconazole. Thus, it can be assumed that the threshold for taste-related adverse events with filapixant is somewhere between 200 and 400 µg/L.

Based on the observed magnitude of the interaction, a filapixant dose adaptation or exclusion of strong CYP3A4/P-gp inhibitors (at least for long term co-administration) is suggested if the compound is progressed into further development. Otherwise, the risk of taste-related side effects is expected to substantially increase as filapixant plasma concentrations will be reached that have been shown previously to lead to a high frequency of taste-related side effects.

Limitations of the study are the relatively small sample size, the single dose treatment of filapixant, the fixed sequence of treatments, and that only healthy young male individuals were included in the study for safety reasons. Those limitations were considered acceptable for the following reasons. The limited sample size is offset by the well-controlled study conditions that allow to reduce variability and thus to obtain an accurate estimate of the interaction magnitude suitable to make recommendations for subsequent studies. A single dose of filapixant was considered meaningful, considering the dose proportional and time linear PK of filapixant [3], which allows to predict multiple dose effects based on single dose data. The fixed sequence design was chosen based on operational considerations. Considering the limited study length and that PK data are not subject to placebo effects, this was considered acceptable. The restriction of the study population to males only was considered necessary since no reproduction toxicity data were available at the time of study start. On the other hand, it is unlikely that the inclusion of women into the study would have led to relevantly different results, considering the known abundance of CYP3A4 in males and females.

## 4. Materials and Methods

### 4.1. In Vitro Studies Investigating Metabolism and Metabolizing Enzymes of Filapixant

#### 4.1.1. Biotransformation of Filapixant in Human Liver Microsomes and Hepatocytes

[^3^H]filapixant was incubated with liver microsomes of different animal species and men as well as hepatocytes suspension of men, rat, mouse, dog, rabbit, and monkey at a concentration of 1 µM (Appendix C). The incubations were analyzed by high-performance liquid chromatography (HPLC) with tandem mass spectroscopy (MS/MS) followed by off-line radioactivity detection to generate metabolite profiles and elucidate or confirm the structures of the metabolites formed.

#### 4.1.2. CYP Phenotyping Studies in Human Hepatocytes, Liver Microsomes, and Recombinant Human CYP Enzymes

The overall turnover of [^3^H]filapixant and formation of individual metabolites was investigated using incubations with human liver microsomes and hepatocytes (Appendix C), and recombinant human CYP isoforms (CYP1A1, 1A2, 1B1, 2A6, 2B6, 2C8, 2C9, 2C18, 2C19, 2D6, 2E1, 2J2, 3A4, 3A5, 3A7, 4A11, 4F2, 4F3A, 4F3B, 4F12, and 19A1 [aromatase]) (Table 1) at 1 µM and with CYP1A1, CYP2D6, CYP2J2, and CYP3A4 in addition at 0.1 µM. Incubations of 1 µM [^3^H]filapixant were performed in human liver microsomes in the presence or absence of CYP isoform–selective inhibitors. Drug depletion and formation of metabolites was determined in the presence of strong CYP3A4 inhibitors (itraconazole, mibefradil and azamulin) as well as other CYP isoform–selective inhibitors (1A1:alpha naphtoflavone, 7 hydroxyflavone, 2D6: quinidine, 2J2: HET0016, and telmisartan). Furthermore, 1 µM non-labeled filapixant was incubated in human hepatocytes in the presence and absence of CYP isoform–selective inhibitors, mibefradil and itraconazole. The incubations were analyzed after the fractionated collection by HPLC and posterior radioactivity detection for [^3^H]filapixant and its metabolites. When unlabeled filapixant was used, the incubation samples were analyzed by HPLC-MS/MS. Further details on the in vitro studies investigating metabolism and metabolizing enzymes of filapixant can be found in an online Supplementary Materials.

### 4.2. Clinical Study

The primary objective of the study was to investigate the influence of orally administered itraconazole, a strong CYP3A4/P-gp inhibitor, on the PK of orally administered filapixant. This was achieved by comparing the endpoints AUC and C_max_ of filapixant from both study periods. The other study objective was to investigate the safety and tolerability of filapixant (with/without itraconazole) in healthy male participants by investigating the frequency and intensity of TEAEs in both periods. Spontaneous reported adverse events were recorded as well adverse events that were reported after open-ended and non-leading verbal questioning of the participants to avoid bias. In addition, itraconazole concentrations were measured in plasma at selected time points to confirm that relevant inhibitory plasma levels were achieved.

The study (NCT03789890; URL: https://clinicaltrials.gov/study/NCT03789890; first registration 27 December 2018) was conducted at CRS Clinical Research Services Berlin GmbH in accordance with the ethical principles that have their origin in the Declaration of Helsinki and the International Council for harmonization (ICH) guideline E6: Good Clinical Practice (GCP) and met all local legal and regulatory requirements. The study was approved by the Ethics Committee of the State of Berlin (Ethikkommission des Landes Berlin, Fehrbelliner Platz 1,10707 Berlin, Germany) (protocol code 19431; version 2.0 from 3 December 2018; approval date 17 December 2018). Informed consent was obtained from all subjects involved in the study.

**Figure 5:**
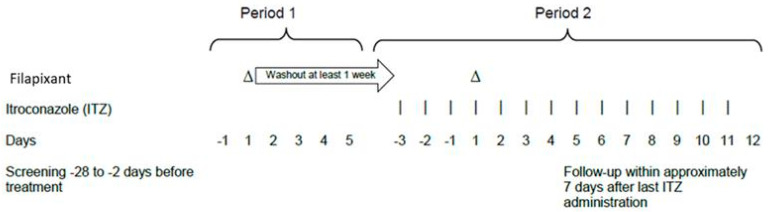
Overall study design (day of filapixant drug administration was defined as day 1; days before this time point were assigned negative numbers).

The study was conducted in a single-center, open-label, fixed sequence design with two periods. In period 1, participants received a single dose of 80 mg filapixant. After a washout period of at least 7 days to ensure the complete elimination of filapixant, period 2 started with a 14-day course of 200 mg itraconazole (Semepra^®^ liquid 10 mg/mL oral solution) once daily. On the 4th day of itraconazole treatment in period 2, participants additionally received a single dose of 80 mg filapixant, the anticipated therapeutic dose, approximately 60 min after itraconazole intake on this day. A 3-day pre-treatment period of itraconazole was chosen to reach steady state itraconazole plasma concentrations at the time of co-administration of filapixant (Figure 5). Administration of filapixant in period 1 and 2 was performed in fasted state.

Healthy male participants, aged between 18 and 55 years and with a body weight of at least 50 kg, were included. Participants’ health was assessed by the investigator (including assessment of medical history, physical examination, blood pressure, pulse rate, 12-lead electrocardiogram, body temperature, and clinical laboratory). Participants with contraindications to itraconazole and participants using any systemic or topically active medication or herbal remedies within 1 week prior to the first drug administration or during the trial until follow-up were excluded. The occasional use of ibuprofen was permissible. A full list of in- and exclusion criteria can be found in Appendix A.

Blood samples for determination of filapixant concentrations were taken from pre-dose until 96 h after administration of filapixant in Period 1 and from pre-dose until 11 days (264 h) after administration of filapixant in Period 2 (filapixant sampling: pre-dose, 0.5, 1, 1.5, 2, 2.5, 3, 4, 6, 8, 12 15, 24, 36, 48, 96, 120, 144, 168, 192, 216, 240, and 264 h after drug administration; itraconazole sampling: before doses 1, 2, 3, 4, 6, 8, 12; 1, 2, and 4 h after the 4th dose and 24 h after the 14th, i.e., last, dose). Concentrations of filapixant and itraconazole were determined according to a method described before [3,8]. The method validation and analysis of the study samples were performed in compliance with the Standard Operating Procedures (SOPs) based on the EMA Guideline on Bioanalytical Method Validation, the FDA Guideline on Bioanalytical Method Validation, the Reflection Paper for Laboratories that Perform the Analysis or Evaluation of Clinical Trial Samples, and the regulations in Good Laboratory Practice (GLP) and GCP, respectively [9,10,11,12,13].

The calibration range was from 0.100 µg/L (LLOQ) to 200 µg/L (upper limit of quantification (ULOQ)) for filapixant and from 1.00 µg/L (LLOQ) to 1000 µg/L (ULOQ) for itraconazole. Accuracy (calculated as percent of nominal) and precision (CV) are as shown in Table 4.

All samples were stored at −20 °C and analyzed within 82 days after sample collection. The available stability data indicated that the analytes are stable for this time period.

Safety and tolerability were assessed based on the frequency and severity of TEAEs. Other safety assessments included standard clinical laboratory tests, vital signs, electrocardiograms, and physical examinations.

PK parameters were calculated by a non-compartmental analysis using the program WinNonlin version 5.3 (Pharsight Corporation, St. Louis, MO, USA), with the Automation Extension (version 2.90, Bayer Pharma AG, Wuppertal, Germany). The statistical evaluation was performed by using the software package SAS release 9.2 (SAS Institute Inc., Cary, NC, USA). All analyses were exploratory. Therefore, no multiplicity adjustments were performed.

The main PK metrics AUC and C_max_ were statistically analyzed, assuming log-normally distributed data. Mean differences between treatments (and their standard error) were determined for logarithmized PK metrics. A total of 90% CIs for treatment differences were determined as well, applying standard methodology for log-normally distributed data. Point estimates and exploratory 90% CIs for the ratios “filapixant + itraconazole/filapixant only” were calculated by re-transformation of the logarithmic data. Only participants providing a valid PK profile from both study periods were to be included in the primary analysis.

This was an exploratory study, and no formal statistical sample size estimation has been performed. However, a sample size of 14 participants was considered sufficient even in a scenario with high intraindividual variability of 30% to estimate the interaction effect with sufficient precision, i.e., that the 90% CIs lies within the range of 83–120% of the point estimate. This accuracy allows us to make recommendations for dosing of filapixant in future studies.

## 5. Conclusions

- Metabolism by CYP3A4 was found to be the main elimination pathway for filapixant in vitro. Drug depletion and formation of metabolites were inhibited in the presence of strong CYP3A4 inhibitors.
- Filapixant was found to be a moderately sensitive substrate of CYP3A4/P-gp. Single oral dosing of 80 mg filapixant concomitantly with multiple daily oral doses of 200 mg itraconazole, a strong CYP3A4/P-gp inhibitor, led to a 1.89-fold higher C_max_ and 4.01-fold higher AUC of filapixant compared to the exposure without itraconazole co-administration. Additionally, the geometric mean terminal half-life (t_1/2_) of filapixant after a single-dose administration was prolonged by co-administration with itraconazole. The changes in both C_max_ and half-life, together with the overall change in AUC, indicate that both clearance and bioavailability were affected by co-administration of itraconazole.
- Single dose oral administration of 80 mg filapixant, with and without itraconazole, was well tolerated by all participants.
- For subsequent studies, the co-administration of filapixant at therapeutic doses with strong inhibitors of CYP3A4/P-gp should be avoided, or an appropriate dose adjustment needs to be implemented.

## Figures and Tables

**Table 1:** Filapixant—remaining drug and formation of metabolites [% of radioactivity] after incubation of 1 µM [^3^H]filapixant with human recombinant CYP Isoforms (Supersomes^TM^) for 60 min.

	Filapixant	M-1 ^a^	M-2	M-3	M-4	M-5 ^a^	M-6	M-7	M-8	M-9	M-11 ^a^
Enzyme	Peak Area [% of Radioactivity]										
Reference	97.1	-		-	-	-	-	1.3	-	-	-
CYP1A1	49.2	-	12.8	2.9	6.5	-	0.5	17.1	0.7	1.2	
CYP1A2	96.9		0.5	-	-	-	-	1.2	-	-	-
CYP1B1	97.0	0.9	-	-	-	-	-	1.1	-	-	-
CYP2A6	97.4	-	-	-	-	-	-	1.0	-	-	-
CYP2B6	97.2	-	-	-	-	-	0.4	1.0	-	-	-
CYP2C8	97.0	-	0.3	-	-	-	-	1.0	-	-	-
CYP2C9	96.3	-	0.7	-	-	0.2	-	1.0	0.2	-	-
CYP2C18	96.1	-	0.5	-	-	-	-	1.0	-	-	-
CYP2C19	92.0	-	2.0	-	-	-	-	2.3	0.2	0.9	-
CYP2D6	86.0	0.8	6.6	5.4	-	-	-	0.8	-		-
CYP2E1	97.8	-	-		-	-	-	0.8	-		-
CYP2J2	72.7	1.1	19.4	0.9	-	-	-	1.1	0.7	1.8	-
CYP3A4	37.9	1.5	15.5	0.5	6.1	0.4	6.0	7.0	3.4	9.2	1.9
CYP3A5	84.8	1.0	1.7	-	1.5	-	0.8	5.3	-	1.9	-
CYP3A7	96.6	1.1	0.5	-	-	-	-	1.2	-	-	-
CYP4A11	97.8	-	-	-	-	-	-	0.6	-	-	-
CYP4F2	97.0	0.9	-	-	-	0.5	-	1.0	-	-	-
CYP4F3A	97.4	0.9	-	-	-	-	-	1.0	-	-	-
CYP4F3B	97.6	-	-	-	-	-	-	1.0	-	-	-
CYP4F12	97.5	-	-	-	-	-	-	1.2	-	-	-
Aromatase	97.7	-	0.2	-	-	-	-	0.7	-	-	-
Reductase	97.8	-	-	-	-	-	-	0.8	-	-	-
insect cell control	97.9	0.8	-	-	-	-	-	0.7	-	-	0.2

^a^ = metabolite, peak area < 2% not presented, peak area < 3.5% of radioactivity is not reported if metabolite could not be assigned.

**Table 2:** PK metrics of filapixant following oral administration of 80 mg filapixant alone and in combination with itraconazole (geometric mean/% coefficient of variation (CV)/range).

PK Metric (unit)	Treatment	n	Geom. Mean	Geom. CV (%)	Min	Median	Max
AUC (µg·h/L)	filapixant	14	695	36.4	402	715	1190
	filapixant + itraconazole	14	2790	22.1	1930	2840	3790
AUC_0-tlast_ (µg·h/L)	filapixant	14	687	37.4	388	713	1180
	filapixant + itraconazole	14	2780	22.2	1930	2840	3790
C_max_ (µg/L)	filapixant	14	90.8	27.6	49.6	93.4	140
	filapixant + itraconazole	14	172	24.3	122	170	261
t_1/2_ (h)	filapixant	14	12.1	14.5	9.52	12.2	14.5
	filapixant + itraconazole	14	22.8	20.2	16.8	22.9	35.2
t_max_ (h)	filapixant	14			1.00	1.00	2.00
	filapixant + itraconazole	14			1.00	1.25	4.02

**Table 3:** Point estimates and 90% CIs for main PK metrics of “filapixant + itraconazole” treated participants vs. “filapixant only” treated participants.

Ratio	PK Metric	N	Geom. CV (%)	Least Square Mean Ratio	90% CI
Filapixant + itraconazole/ Filapixant only	AUC	14	13.61	4.01	3.66; 4.39
	C_max_	14	19.07	1.89	1.67; 2.15

**Table 4:** Bioanalytical method performance for measurements of filapixant and itraconazole.

**Filapixant**	
Calibration standards mean inter-assay accuracy of back-calculated concentrations	99.50% to 100.50%
Calibration standards precision	≤4.13%
Accuracy at the lowest calibration standard (LLOQ)	99.90%
Precision at the lowest calibration standard (LLOQ)	3.67%
Concentration range of Quality control (QC) samples (μg/L)	0.300 to 150
QC accuracy	94.33% to 98.40%
QC precision	2.21% to 4.35%
**Itraconazole**	
Calibration standards mean inter-assay accuracy of back-calculated concentrations	98.00% to 102.00%
Calibration standards precision	≤5.82%
Accuracy at the lowest calibration standard (LLOQ)	99.90%
Precision at the lowest calibration standard (LLOQ)	2.59%
Concentration range of QC samples (μg/L)	3.00 to 750
QC accuracy	90.40% to 96.33%
QC precision	1.87% to 5.16%

## Data Availability

Availability of the data underlying this publication will be determined according to Bayer’s commitment to the European Federation of Pharmaceutical Industries and Associations and Pharmaceutical Research and Manufacturers of America principles for responsible clinical trial data sharing, pertaining to scope, time point, and process of data access. Bayer commits to sharing upon request from qualified scientific and medical researchers, patient-level clinical trial data, study-level clinical trial data, and protocols from clinical trials in patients for medicines and indications approved in the USA and the European Union as necessary for performing legitimate research. This commitment applies to data on new medicines and indications that have been approved by the European Union and US regulatory agencies on or after 1 January 2014. Interested researchers can use www.clinicalstudydatarequest.com to request access to anonymized patient-level data and supporting documents from clinical studies to do further research that can help advance medical science or improve patient care. Information on the Bayer criteria for listing studies and other relevant information is provided in the study sponsors section of the portal. Data access will be granted to anonymized patient-level data, protocols, and clinical study reports after approval by an independent scientific review panel. Bayer is not involved in the decisions made by the independent review panel. Bayer will take all necessary measures to ensure that patient privacy is safeguarded.

## Supplementary Materials

The following supporting information can be downloaded at: https://www.mdpi.com/article/10.3390/molecules30204110/s1.

## Abbreviations

The following abbreviations are used in this manuscript:

ABT	1-Aminobenzotriazol
CI	Confidence interval
CL_int_	Intrinsic clearance
CV	Coefficient of variation
CYP	Cytochrome P450
CYP3A4	Cytochrome P450 3A4
GCP	Good Clinical Practice
GLP	Good Laboratory Practice
HPLC	High-performance liquid chromatography
ICH	International Council for Harmonization
LLOQ	Lower limit of quantification
MS/MS	Tandem mass spectrometry
P-gp	P-glycoproteinP
PK	Pharmacokinetics
QC	Quality control
SD	Standard deviation
TEAE	Treatment-emergent adverse event
ULOQ	Upper limit of quantification
**Definitions of Pharmacokinetic Terms**	
AUC	Area under the concentration–time curve from zero to infinity after single dose
AUC_0 tlast_	AUC from time zero to last data point above LLOQ
C_max_	Maximum observed drug concentration after single dose administration
t_1/2_	Half-life associated with the terminal slope
t_max_	Time to reach C_max_ after single dosing

## Appendix A. In- and Exclusion Criteria

### Appendix A.1. The Inclusion Criteria

1. The informed consent must be signed before any study specific tests or procedures are performed.
2. Ability to understand and follow study-related instructions.
3. Participant must be 18 to 55 years of age (inclusive), at the time of signing the informed consent.
4. Male.
5. Participant is healthy as determined by the investigator (including assessment of medical history, physical examination, blood pressure, pulse rate, 12-lead electrocardiogram, body temperature, and clinical laboratory).
6. Confirmation of the participant’s health insurance coverage at screening.
7. Body mass index (BMI) above or equal 18 and below or equal 30.0 kg/m^2^ at screening.
8. Body weight of at least 50 kg at screening.
9. Agreement by the participant that he and his female partner of childbearing potential will use an accepted method of contraception for the duration of the study. Note: The parallel application of two of the following methods of contraception is considered adequately reliable:

Mechanical barrier (condom for the participant), in addition to hormonal contraception of the female partner (oral contraception with a Pearl index <1, hormone patch, hormone ring, hormone spiral, three-month depot injection or implants), or copper spiral or sterilized (tubal ligation, hysterectomy) female partner.

Contraception requirements apply from first dosing until 90 days after the last study intervention.

### Appendix A.2. Exclusion Criteria

1. History or evidence of any clinically significant cardiovascular, gastrointestinal, endocrine, hematologic, hepatic, immunologic, metabolic, urologic, pulmonary, neurologic, dermatologic, psychiatric, renal, and/or other major disease or malignancy, as judged by the investigator.
2. Clinically relevant history of allergic conditions (including drug allergies, asthma, eczema, or anaphylactic reactions, but excluding untreated, asymptomatic, seasonal allergies at the time of dosing).
3. Clinically relevant abnormalities in the medical examination (including medical history, physical examination, vital signs, laboratory tests, and electrocardiogram) as judged by the investigator at screening or upon admission to the clinical unit.
4. Participant has/had febrile illness or symptomatic, viral, bacterial (including upper respiratory infection), or fungal (non-cutaneous) infection within 1 week prior to admission to the clinical unit.
5. History or current condition for which it can be assumed that the absorption, distribution, excretion and effect of the study interventions will be influenced., e.g., history of malabsorption, esophageal reflux, peptic ulcer disease, erosive esophagitis frequent, or surgical interventions including cholecystectomy, impaired renal function.
6. Any malignant tumor and history thereof.
7. Acute or chronic progressive liver diseases, e.g., disturbances of the bilirubin excretion (Dubin-Johnson and Rotor syndromes); disturbances of the bile secretion and the flow (cholestasis); presence or history of liver tumors (benign or malignant); between the subsidence of a viral hepatitis (normalization of liver parameters) and the screening for this study there must be an interval of at least six months.
8. Clinically relevant kidney diseases (e.g., glomerulonephritis), or renal injury associated with multisystem diseases/disorders (e.g., systemic lupus erythematosus, diabetic nephropathy); severe renal insufficiency or acute renal failure; a history of a single episode of uncomplicated nephrolithiasis does not prevent participation.
9. Known or suspected allergy or hypersensitivity, to filapixant, to itraconazole, or any of their excipients.
10. Contraindications to itraconazole (symptoms or history of ventricular dysfunction, heart failure, liver disease).
11. History of alcohol or drug abuse within two years prior to screening.
12. Any use of systemic or topically active medication or herbal remedies, prescription or non-prescription, within one week prior to the first study drug administration or during the trial until follow-up (occasional use of ibuprofen is permissible). Particularly, this includes drugs that might affect the PK of filapixant, e.g., laxatives, loperamide, metoclopramide, antacids, H_2_-receptor antagonists, CYP3A4 inhibitors, or inducers.
13. Regular use of alimentary supplements, e.g., carnitine products, anabolics, high-dose vitamins.
14. Not able or willing to abstain from consumption of the following:
  - Caffeine containing beverages or food (e.g., tea, coffee, cola, chocolate, mate) from 24 h before each filapixant administration and on the PK profile days (Day 1 of Period 1 and 2)
  - Quinine containing beverages or food (bitter lemon, tonic water) from 1 week before the first study intervention administration until the last PK blood sample
  - Food and beverages containing furanocoumarin derivatives (e.g., grapefruit, pomelos, limes, Seville oranges) from 1 week before the first study intervention administration until the last PK blood sample
  - Poppy seeds containing food from 1 week before the first study intervention administration until last PK blood sample
15. History of drinking more than 14 units of alcohol per week (1 unit = 10 g pure alcohol), i.e., on average approximately 20 g/day, or 400 mL of beer [5%], or 60 mL of spirits [35%], or 170 mL of wine [12%]) within three months prior to admission to the clinical unit.
16. Smoking more than 10 cigarettes per day, and not willing or able to abstain from smoking from 10 h before until 8 h after filapixant administration.
17. Significant blood loss, e.g., by donation of more than 100 mL of whole blood or plasma within four weeks or 500 mL whole blood within three months before the first study intervention administration until follow-up.
18. Plasmapheresis within four weeks prior to screening and until the follow-up visit.
19. PQ > 220 ms, QT interval corrected using Bazett’s formula (QTcB) > 450 ms, QRS >120 ms.
20. Pulse rate <50 or >90 bpm (a lower heart rate between 50 bpm and 45 bpm is acceptable in case of normal thyroid function and absence of symptoms of bradycardia), systolic blood pressure <100 or >140 mmHg, and blood pressure <60 or >90 mmHg.
21. Clinically relevant findings in the physical examination.
22. Poor venous access.
23. Clinically relevant abnormalities in the laboratory parameters at screening and upon admission to the clinical unit. In particular:
  - ALT, aspartate aminotransferase (AST), or Bilirubin outside normal ranges;
  - Thyroid stimulating hormone (TSH) > the ULN;
  - Positive results for hepatitis B virus surface antigen (HBsAg), hepatitis C virus antibodies (anti-HCV), and human immune deficiency virus antigen-/antibody (anti-HIV 1 + 2).
24. Positive urine drug screening.
25. Positive alcohol breath test.
26. Previous assignment to study intervention during this study.
27. Previous (within two months prior to administration) or concomitant participation in another clinical study.
28. Participant is in custody by order of an authority or a court of law.
29. Close affiliation with the investigational site, e.g., a close relative of the investigator or a dependent person (e.g., employee or student of the investigation site).
30. Participant is an employee of Bayer AG.
31. Criteria which in the opinion of the investigator preclude participation for scientific reasons, for reasons of compliance, or for reasons of the participant’s safety.

## Appendix C. Human Liver Microsome and Hepatocyte Data

**Table A1:** Recovery of filapixant and metabolite formation of [^3^H]filapixant in human liver microsomes and hepatocytes.

Filapixant/Metabolite	Liver Microsomes ^a,b^	Hepatocytes ^c^
Filapixant	45	68–87
M-1	1.8	2.6–3.6
M-2	15	1.7–7.6
M-3	0.9	0.4–0.8
M-4	7.6	1.0–7.5
M-5	n.d.	0–0.2
M-6	4.4	n.d.
M-7	9.1	2.3–3.2
M-8	2.4	0–1.7
M-9	6.7	0–1.3
M-10		0.2–0.9
M-11		0–0.3

Amounts < 0.5% were detected by LC-MS; < 0.5% of radioactivity is not listed, ^a^ Data are for 1 h of incubation, ^b^ n = 1 because pooled liver microsomes were used, ^c^ Data are the range in three batches for 4 h of incubation.
