# Supplementary material for: Investigation of the Relevance of CYP3A4 Inhibition on the Pharmacokinetics of the Novel P2X3 Antagonist Filapixant: Results of In Vitro Explorations and a Fixed-Sequence Clinical Trial with Itraconazole in Healthy Volunteers
Source: Int J Mol Sci. 2025 Oct 20;26(20):10177. doi: 10.3390/ijms262010177 (PMC12564180; doi:10.3390/ijms262010177)

Supplement to: Investigation of the relevance of CYP3A4 inhibition on the pharmacokinetics of the novel P2X3 antagonist filapixant – results of in vitro explorations and clinical study with itraconazole

K Francke, A. Rottmann, S Klein, J. Hoechel, C Friedrich

For author affiliations please see the main paper.

Biotransformation of Filapixant in Human Liver Microsomes and Hepatocytes

Human liver microsomes XTreme 200, lot No. 1210223 (pool, 20 mg/mL protein) were purchased from XenoTech LLC (Lenaxa, KS, USA). Human liver cytosol H0610.C, lot. 1310087 (pool, protein) was purchased from XenoTech LLC. Cryopreserved human hepatocytes were obtained from Bioreclamation IVT (former Celsis In Vitro Technologies, Baltimore, MD, USA, donor: TWT).

[^{3}H]filapixant from a 0.1 mM stock solution dissolved in acetonitrile was incubated at 1 µM with 0.5 mg/mL microsomal protein in 100 mM potassium phosphate buffer (pH 7.4) or with 1.0 mg/mL cytosolic protein in 50 mM potassium phosphate buffer (pH 7.4). The incubation mixture for both microsomes and cytosol also contained an NADPH-generating system (1.2 mM NADP, 8 mM glucose 6-phosphate, 1 U/mL glucose 6-phosphate dehydrogenase, 38 mM KCl, and 5 mM $MgCl_2$ solution). Liver microsomes and cytosol were incubated at 37 °C for 1 hour (microsomes) or for 0, 4, and 24 hours (cytosol). Incubations without NADPH were performed as negative controls.

[^{3}H]filapixant from a 0.1 mM or 1mM stock solution dissolved in acetonitrile was incubated at 1 µM with human primary hepatocytes (suspension culture) in William's E Medium buffer at 37 °C for 4 hours.

Radioactivity of liquid samples (in vitro incubations stopped with acetonitrile [30% v/v]) was measured by liquid scintillation counting in a liquid scintillation spectrometer Tri-Carb® 2900 (Perkin Elmer, Boston, MA, USA) with automatic quench correction by the external standard channel ratio method at 17 °C using Pico Fluor as a scintillation cocktail.

Samples were analyzed by high-performance liquid chromatography (HPLC) with off-line radioactivity monitoring of timed fractions. Simultaneously, on-line mass spectrometry (MS) was performed using the Orbitrap XL or Orbitrap Fusion Lumos mass spectrometer (Thermo Fisher, Bremen, Germany). A small part of the HPLC-eluent was split directly into the mass spectrometer. The major part of the HPLC-eluent was collected onto LumaPlate™-96-well plates (Perkin Elmer, Boston, MA, USA) with the Fraction Collector (Bayer, Leverkusen, Germany) after HPLC separation. The radioactivity in each well was detected by the Scintillation Counter Topcount NXT (Perkin Elmer, Boston, MA, USA) to generate a radio chromatogram. In addition, liquid samples were analyzed without radioactivity detection for HPLC-MS and ultraviolet detection. Exact mass determined by high resolution MS and mass changes in comparison with the parent drug in combination with the fragment pattern from tandem and sequential MS were used to propose the structures of metabolites or confirm structures by comparison with reference compounds.

HPLC and MS data were stored and evaluated using Xcalibur® software (Thermo Fischer, Dreieich, Germany). Liquid scintillation data were stored in a worksheet and evaluated using Wallac-TopCount Connector software (Version 3.0.3.56; Bayer Business Services GmbH, Leverkusen, Germany).

CYP Phenotyping Studies in Human Hepatocytes and Liver Microsomes, and Recombinant Human CYP Enzymes

Pooled human liver microsomes (XTreme200, lot 1010420 and lot 1210223, 20 mg/mL) were purchased from XenoTech LLC. Cryopreserved human hepatocytes were obtained from Bioreclamation IVT for donor WSS.

Incubation of [3H]filapixant with human liver microsomes and recombinant human CYP enzymes

[3H]filapixant from a stock solution dissolved in acetonitrile was incubated at 1 µM with 0.5 mg/mL liver microsomal protein in 50 mM potassium phosphate buffer (pH 7.4) with 1 mM EDTA and an NADPH-regenerating system (1 mM NADP, 10 mM glucose 6-phosphate, and 1 U/mL glucose 6-phosphate dehydrogenase) at 37 °C. In addition to human liver microsomes, recombinant CYP isoforms (Supersomes™: CYP1A1, 1A2, 1B1, 2A6, 2B6, 2C8, 2C9, 2C18, 2C19, 2D6, 2E1, 2J2, 3A4, 3A5, 3A7, 4A11, 4F2, 4F3A, 4F3B, 4F12, and 19 aromatase) were used at a final concentration of 50 pmol CYP/mL. Microsomal preparations from insect cells without and with reductase were used for control experiments. The reaction was initiated by the addition of filapixant after 5 minutes pre-incubation at 37 °C.

Incubation of unlabeled filapixant with human liver microsomes or hepatocytes in the presence or absence of CYP-isoform selective inhibitors

Unlabeled filapixant from a 0.1-mM stock solution dissolved in acetonitrile was incubated at 1 µM with pooled human liver microsomes (1.0 mg/mL) in a total volume of 0.3 mL in the absence and presence of CYP isoform-selective inhibitors. Incubation mixtures contained 50 mM potassium phosphate buffer (pH 7.4) with 1 mM EDTA, and an NADPH regenerating system (1 mM NADP, 10 mM glucose 6-phosphate, and 1 U/mL glucose 6-phosphate dehydrogenase). Direct-acting CYP-isoform-selective inhibitors (7-hydroxyflavone for CYP1A1, danazol for CYP2J2, montelukast for CYP2C8, sulfaphenazole for CYP2C9, benzylphenobarbital for CYP2C19, and quinidine for CYP2D6) were incubated simultaneously with filapixant. Mechanism-based inhibitors

(mibefradil and azamulin for CYP3A4; aminobenzotriazole as pan-CYP-inhibitor) were pre-incubated with the human liver microsomes in the above incubation mixture at 37 °C for 15 minutes before the reaction was initiated by addition of filapixant and incubation continued for 60 minutes.

Unlabeled filapixant from a 0.1 mM stock solution dissolved in acetonitrile was incubated at 1 µM with human hepatocytes (donor WSS, suspension culture) in Williams' E Medium at 37 °C for 4 hours in the absence and presence of CYP isoform-selective inhibitors. The CYP2J2 inhibitors danazol and telmisartan were incubated simultaneously with filapixant. Mibefradil, azamulin, and aminobenzotriazole were pre-incubated in hepatocytes at 37 °C for 15 minutes before the reaction was initiated by addition of filapixant and incubation continued for 4 hours.

For both liver microsomes and hepatocytes, incubations with unlabeled filapixant were terminated with acetonitrile (100% v/v) containing a generic internal standard (BAY 1113914), while incubations with [^{3}H]filapixant were terminated without internal standard.

For [^{3}H]filapixant, metabolite profiles were obtained by high-performance liquid chromatography (HPLC) separation of samples with off-line radioactivity detection, as described above. For unlabeled filapixant, analysis of filapixant and its metabolites was performed using HPLC-MS/MS on a Vanquish UPLC coupled via a heated electrospray ionization (HESI) Source to a Q-Exactive Hybrid Quadrupol-Orbitrap mass spectrometer or Orbitrap Fusion Lumos (Thermo Fisher Scientific, Waltham, MA, USA). Identification of metabolites was based on measurement of the exact mass and on MS/MS fragmentation spectra.

Table S1: Metabolite formation of 1 µM [3H]filapixant in pooled human liver microsomes after 60 min incubation in presence and absence of CYP-isoform selective inhibitors

Inhibitor	Concentration [µM]	BAY 1902607	M-1	M-2	M-3	M-4	M-6	M-7	M-8	M-9	M-11	M-13
						[% of control activity]						
Control		-	100.0	100.0	100.0	100.0	100.0	100.0	100.0	100.0	100.0	100.0
7-Hydroxyflavone	2	94.6	133.6	104.5	111.6	49.4	131.9	104.0	119.1	-	140.4	102.3
ABT[a]	1000	5.9	30.7	-	-	-	-	19.2	-	-	-	-
α-Naphthoflavone	1	95.7	89.8	95.6	92.4	106.2	115.3	93.5	109.0	96.8	109.9	101.0
α-Naphthoflavone	5	97.6	169.5	103.6	91.8	113.4	96.1	102.0	93.5	104.3	98.5	81.6
Azamulin[a]	2	8.5	71.8	2.1	-	-	-	25.3	-	-	-	-
HET0016	5	88.6	106.2	98.1	93.6	42.6	149.7	81.2	121.4	67.3	147.1	90.2
Itraconazole	1	23.3	42.2	24.8	29.6	-	18.2	37.4	14.2	24.5	-	12.8
Itraconazole	10	9.0	45.3	7.2	-	-	-	28.7	-	-	-	-
Mibefradil[a]	2	9.7	47.2	4.0	-	-	-	28.7	5.8	7.5	-	-
Quinidine	2	91.9	154.6	91.2	60.9	102.6	109.0	103.6	90.0	102.1	121.5	94.1
Quinidine	10	57.7	110.5	59.5	37.3	58.2	61.9	64.5	24.9	116.1	34.5	24.1
Telmisartan	5	80.9	95.9	91.6	89.7	49.4	111.6	87.5	107.8	66.3	89.1	89.1

a = additionally 15 min preincubation of inhibitor

Table S2: Depletion of 1 µM filapixant in presence of human recombinant CYP1A1, CYP2D6, CYP 2J2 and CYP3A4

Human CYP isoform	Enzyme [pmol CYP/mL]	Time [min]	Concentration drug [µM]	Remaining drug [%]	CL_{int} [µL/min/pmol CYP]
CYP1A1	50	0	1.13	100.0	0.217
		2	1.07	94.8	
		5	1.06	93.6	
		10	0.95	84.0	
		20	0.86	75.5	
		30	0.76	66.6	
		45	0.66	58.0	
		60	0.60	52.6	
CYP2D6	50	0	1.242	100.0	0.0618
		2	1.21	97.4	
		5	1.132	91.1	
		10	1.084	87.3	
		20	1.122	90.3	
		30	1.047	84.3	
		45	1.021	82.2	
		60	1.007	81.1	
CYP2J2	50	0	1.258	100.0	0.126
		2	1.211	96.3	
		5	1.184	94.1	
		10	1.152	91.6	
		20	1.098	87.3	
		30	1.008	80.1	
		45	0.899	71.5	
		60	0.867	68.9	
CYP3A4	25	0	1.249	100.0	1.32
		2	1.124	90.0	
		5	0.873	69.9	
		10	0.669	53.6	
		15	0.447	35.8	
		20	0.317	25.4	
		40	0.235	18.8	
		60	0.171	13.7	

Table S3: Depletion of 0.1 µM filapixant in presence of human recombinant CYP1A1, CYP2D6, CYP 2J2 and CYP3A4

Human CYP isoform	Enzyme [pmol CYP/mL]	Time [min]	Concentration drug [µM]	Remaining drug [%]	CL_{int} [µL/min/pmol CYP]
CYP1A1	50	0	0.103	100.0	0.252
		2	0.102	99.0	
		5	0.099	96.1	
		10	0.094	91.3	
		20	0.079	76.7	
		30	0.067	65.0	
		45	0.057	55.3	
		60	0.051	49.5	
CYP2D6	50	0	0.115	100.0	0.0716
		2	0.112	97.4	
		5	0.112	97.4	
		10	0.107	93.0	
		20	0.104	90.4	
		30	0.101	87.8	
		45	0.096	83.5	
		60	0.092	80.0	
CYP2J2	50	0	0.123	100.0	0.132
		2	0.126	102.4	
		5	0.118	95.9	
		10	0.112	91.1	
		20	0.108	87.8	
		30	0.104	84.6	
		45	0.091	74.0	
		60	0.083	67.5	
CYP3A4	25	0	0.121	100.0	1.66
		2	0.111	91.7	
		5	0.089	73.6	
		10	0.066	54.5	
		15	0.039	32.2	
		20	0.025	20.7	
		40	0.015	12.4	
		60	0.011	9.1	

Table S4: Depletion of 1 µM filapixant in human hepatocytes in absence and presence of CYP3A4 selective (itraconazole, mibefradil) and pan-CYP (ABT) inhibitors

Incubation	Time [min]	Concentration drug [µM]	Remaining drug [µM]	Cl_{int}[a] [µL/min/mio cells]	% of control CL_{int} inhibited
Control	0	0.925	100.0	24.7	n.a.
	15	0.579	62.6		
	30	0.353	38.2		
	45	0.225	24.3		
	60	0.144	15.6		
	90	0.080	8.6		
	120	0.041	4.4		
	240	<LOQ	n.c.		
Itraconazole (2 µM)	0	0.927	100.0	1.39	94.4
	15	0.820	88.5		
	30	0.864	93.2		
	45	0.745	80.4		
	60	0.728	78.5		
	90	0.699	75.4		
	120	0.744	80.3		
	240	0.596	64.3		
ABT[b] (1000 µM)	0	0.924	100.0	1.37	94.5
	15	0.894	96.8		
	30	0.865	93.6		
	45	0.842	91.1		
	60	0.813	88.0		
	90	0.868	93.9		
	120	0.778	84.2		
	240	0.645	69.8		
Mibefradil[b] (2 µM)	0	0.998	100.0	1.10	95.6
	15	1.025	102.7		
	30	1.020	102.2		
	45	0.932	93.4		
	60	0.975	97.7		
	90	0.827	82.9		
	120	0.779	78.1		
	240	0.822	82.4		

a = Cl_{int} values calculated on 30 to 240 min time points
b = additionally 15 min preincubation of inhibitor
n.c. = not calculated

Table S5: Apparent enzyme kinetic parameters obtained after 20 min incubation of filapixant in recombinant human CYP3A4 (25 pmol/mL)

Product	$K_{m,app}$ [µM]	V_{max} [peak area/pmol CYP/min]
M-2	29.5	3.56E+05
M-4	4.7	9.20E+03
M-6	27.6	7.78E+04
M-7	27.7	1.32E+05
M-8	7.7	2.48E+04
M-9	129.6	2.23E+05
Sum of metabolites[a]	30.2	7.25E+05

a = peak area of metabolites M-2, M-4, M-6, M-7, M-8, M-9 was summarized and evaluated for kinetic parameters

Table S6: Apparent enzyme kinetic parameters obtained after 30 min incubation of filapixant in human liver microsomes (0.5 mg/mL)

Product	$K_{m,app}$ [µM]	V_{max} [peak area/pmol CYP/min]
M-2	108.7	9.60E+05
M-4	7.9	1.02E+04
M-6	68.0	1.32E+05
M-7	102.7	4.05E+05
M-8	28.5	4.34E+04
M-9	n.c.	-
Sum of metabolites[a]	129.1	2.46E+06

a = peak area of metabolites M-2, M-4, M-6, M-7, M-8, M-9 was summarized and evaluated for kinetic parameters

Figure S1: Depletion of 1 µM [^{3}H]Filapixant in a panel of human recombinant CYP isoforms after 60 min incubation time

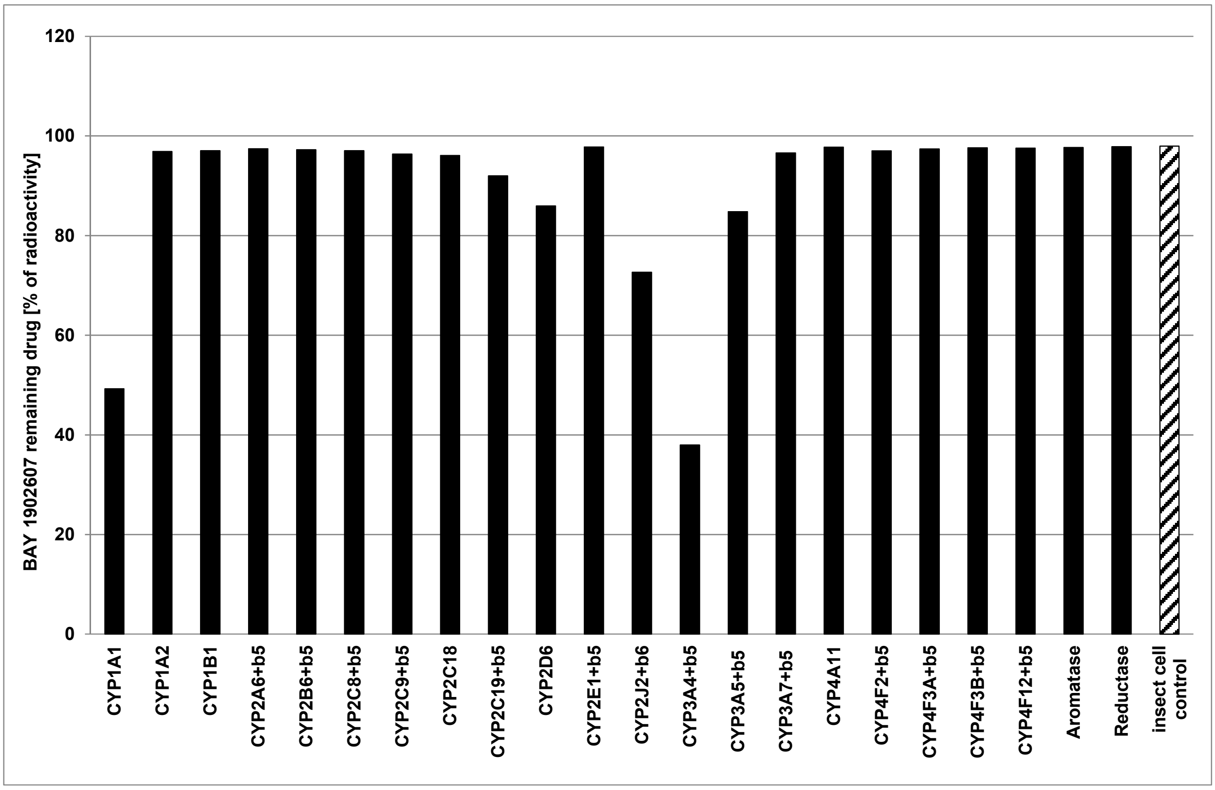

Figure S2:Formation of metabolites from 1 µM [^{3}H]Filapixant in human recombinant CYP isoforms [pmol/(pmol P450 x min)]

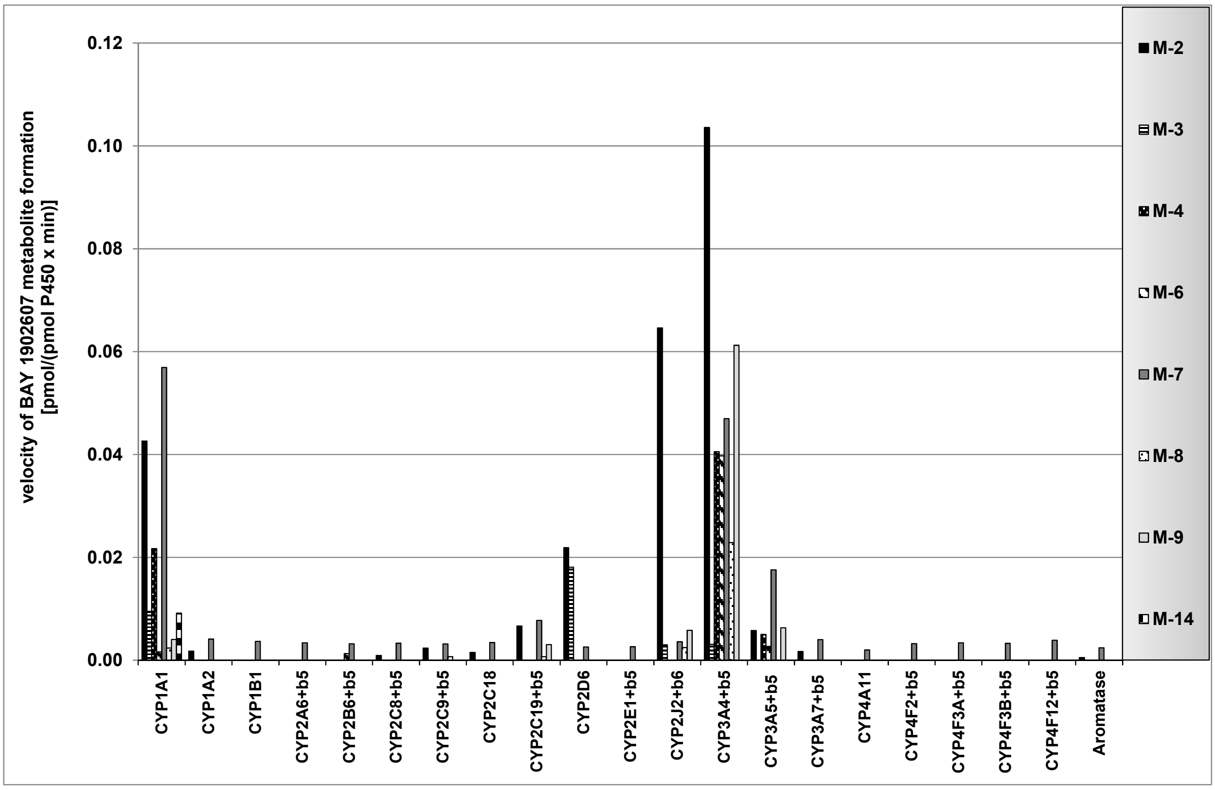

Figure S3: Depletion of 1 µM [^{3}H]Filapixant in pooled human liver microsomes after 60 min incubation in presence of CYP-isoform selective inhibitors

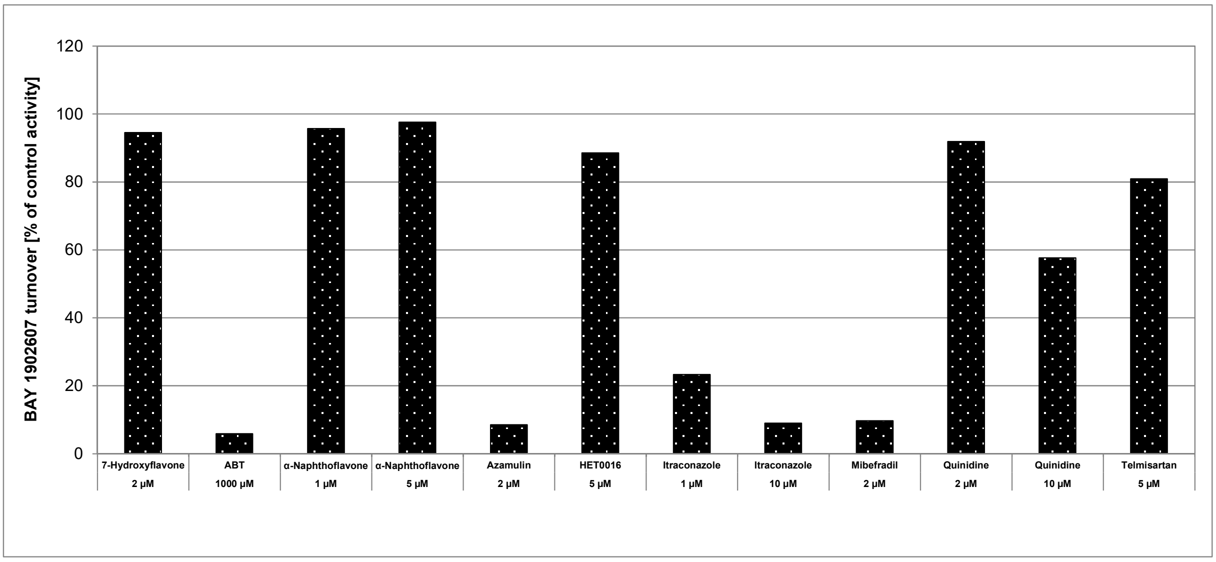

ABT, azamulin and mibefradil were preincubated for 15 min

Figure S4: Metabolite formation of 1 µM [^{3}H]Filapixant incubated in pooled human liver microsomes for 60 min in presence of CYP-isoform selective inhibitors

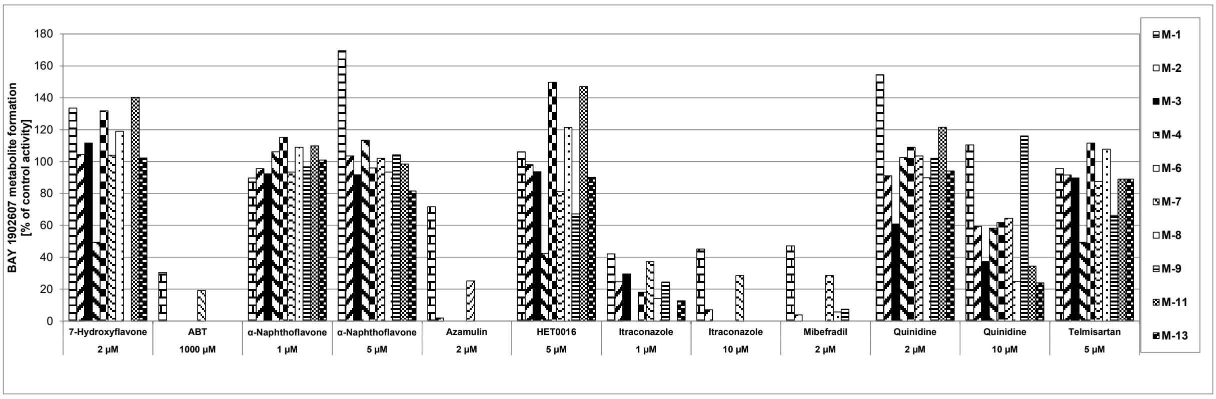

ABT, azamulin and mibefradil were preincubated for 15 min

Figure S5: Depletion of 1 µM filapixant in recombinant CYP1A1, 2D6, 2J2 (50 pmol/mL each) and 3A4 (25 pmol CYP/mL)

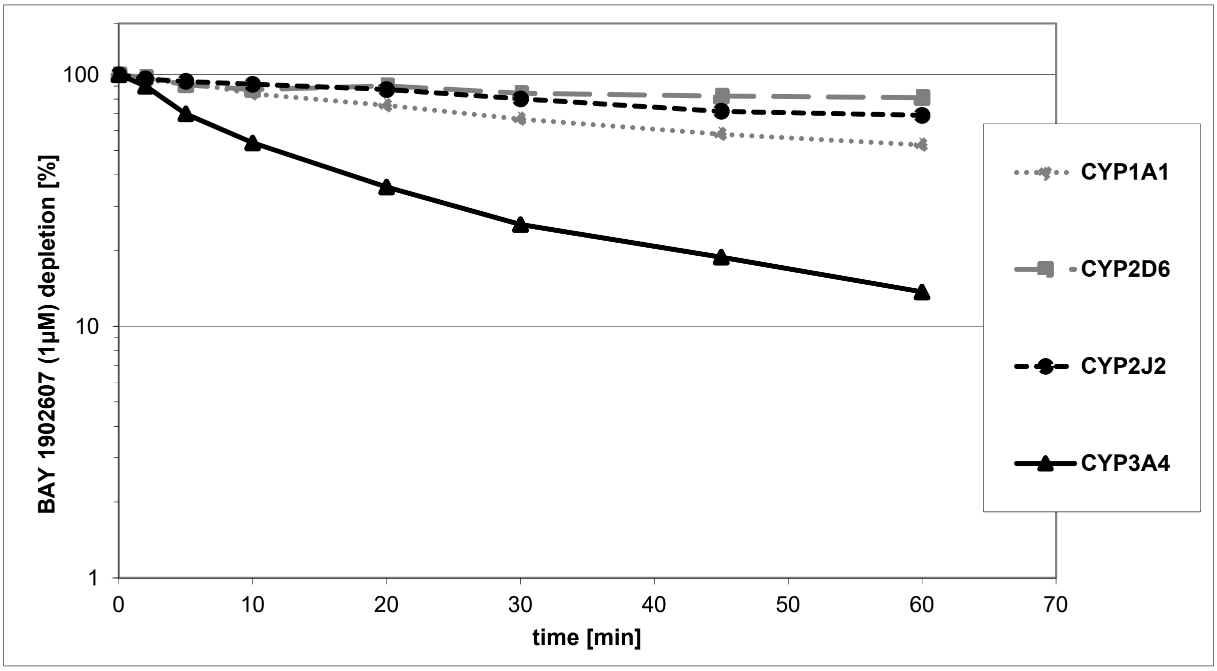

Figure S6: Depletion of 0.1 µM filapixant in recombinant CYP1A1, 2D6, 2J2 (50 pmol/mL each) and 3A4 (25 pmol CYP/mL)

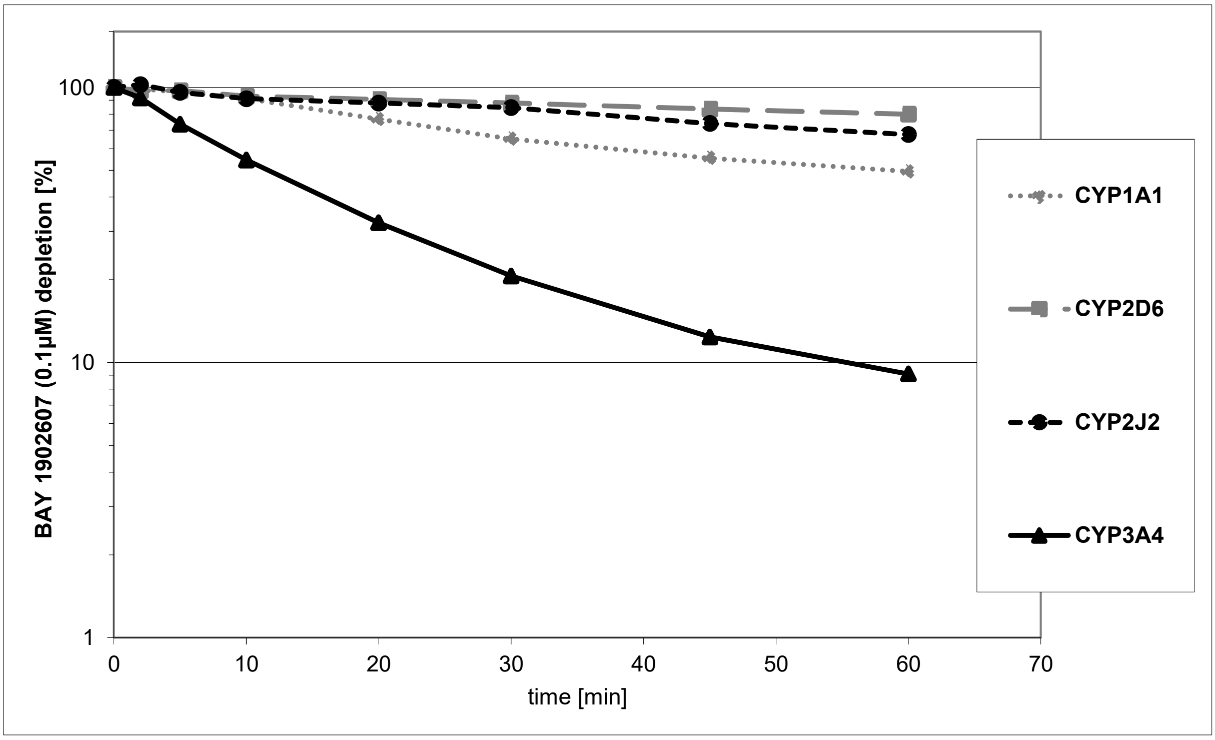

Figure S7: Filapixant: Michaelis-Menten plot for the formation of metabolites M-2, M-4, M-7 and sum of metabolites (M-2, M-4, M-6, M-7, M-8 and M-9) in human recombinant CYP3A4 (25 pmol/mL, incubation time: 20 min)

M-2

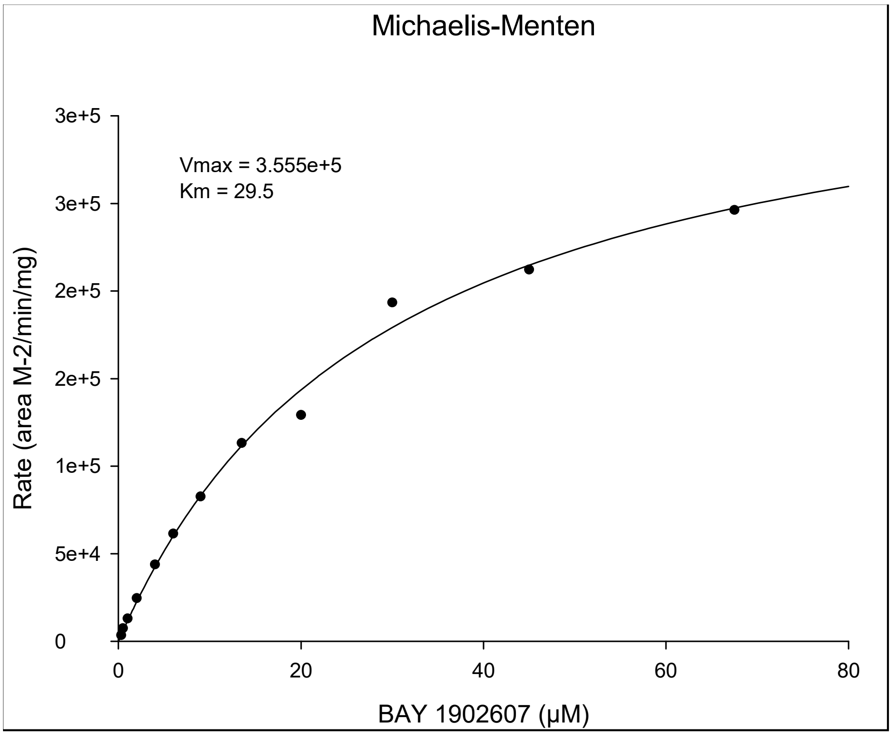

M-4

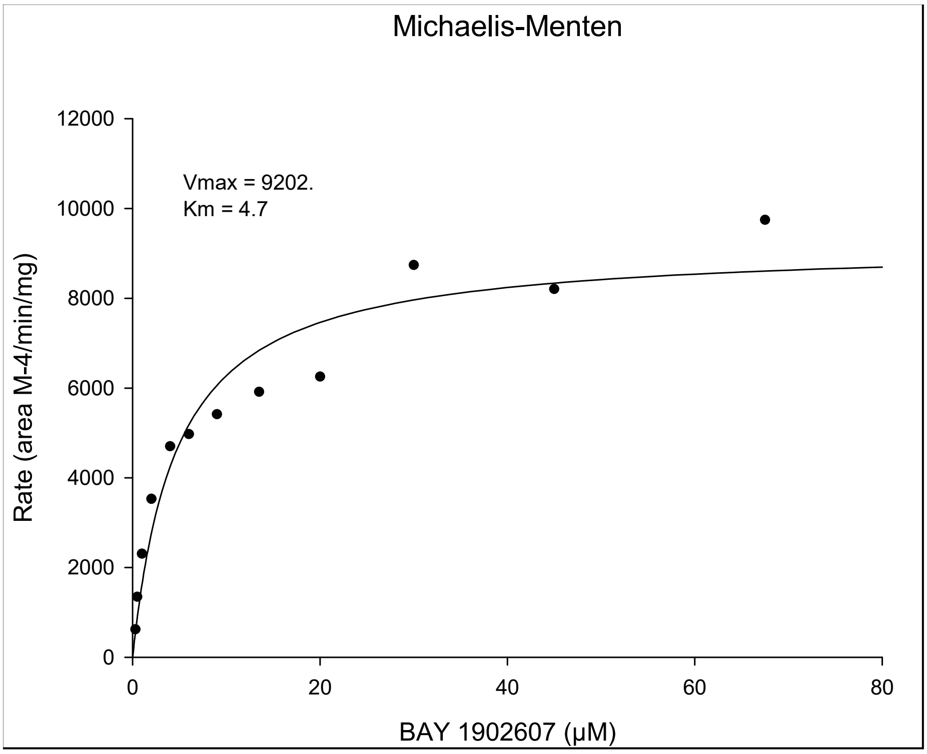

M-7

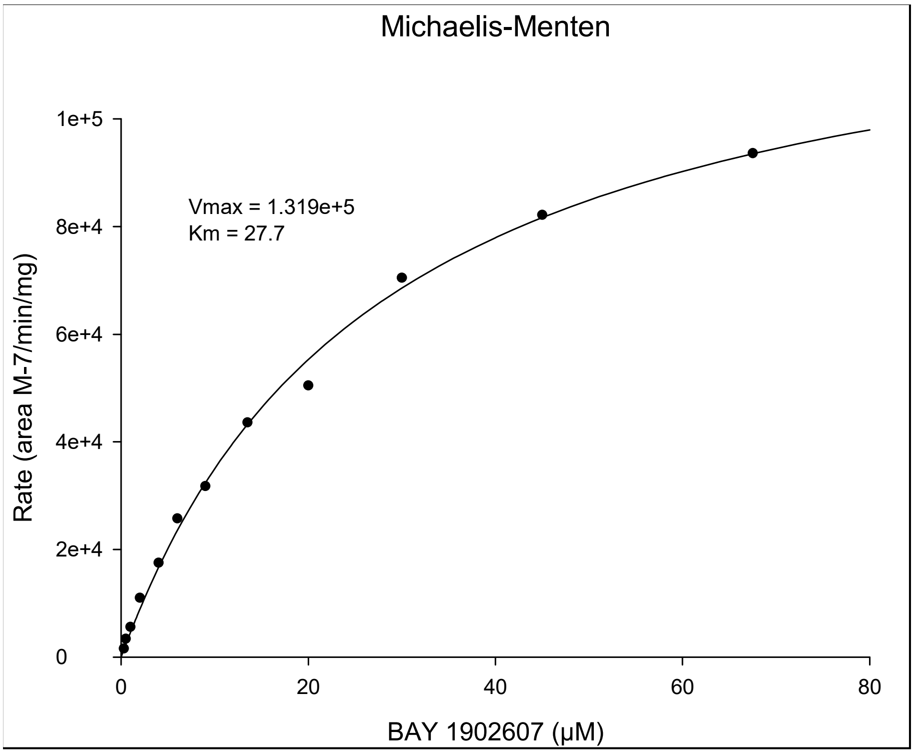

Sum of metabolites M-2, M-4, M-6, M-7, M-8, M-9

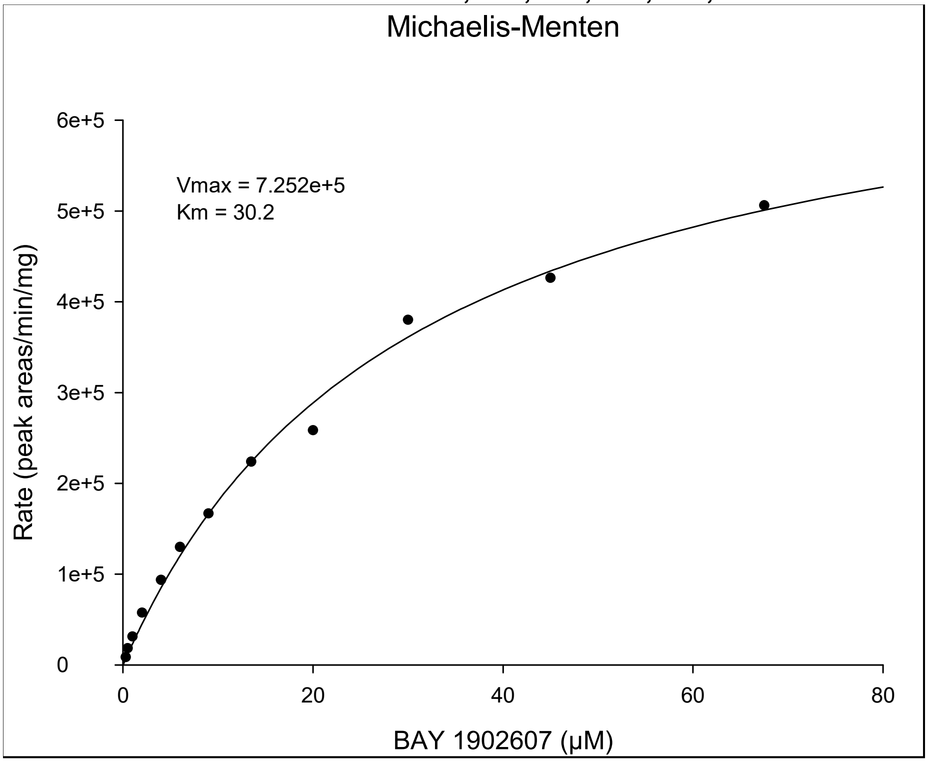

Figure S8: Filapixant: Michaelis-Menten plot for the formation of metabolites M-2, M-4, M-7 and sum of metabolites (M-2, M-4, M-6, M-7, M-8 and M-9) in human liver microsomes (0.5 mg/mL, incubation time: 30 min)

M-2

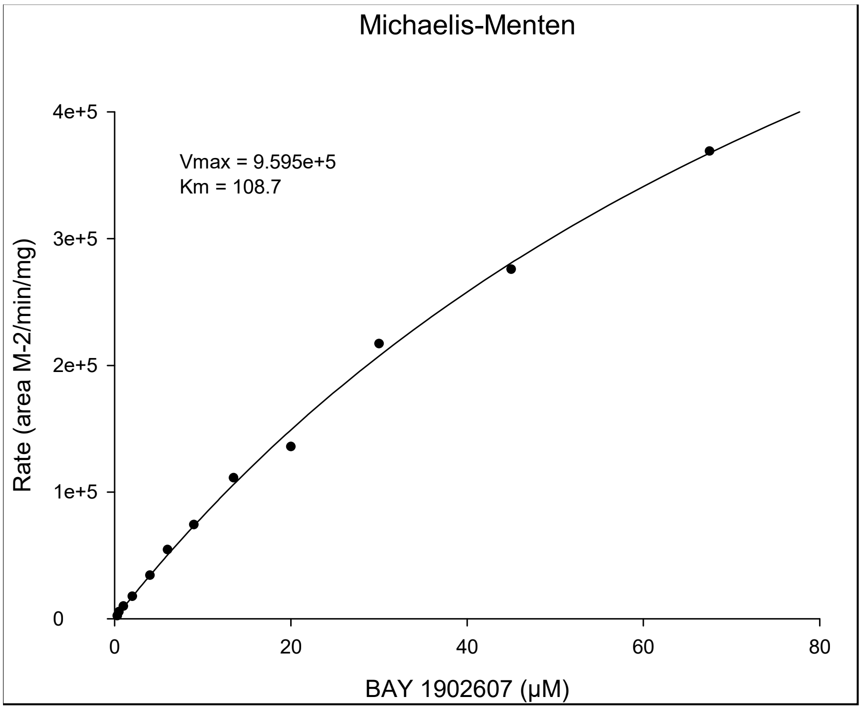

M-4

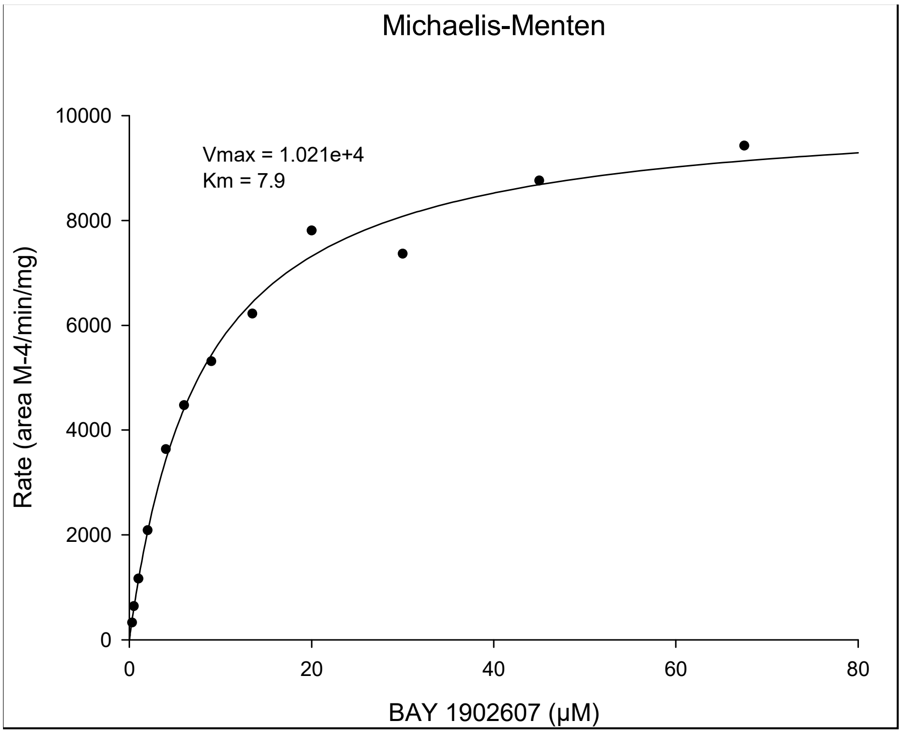

M-7

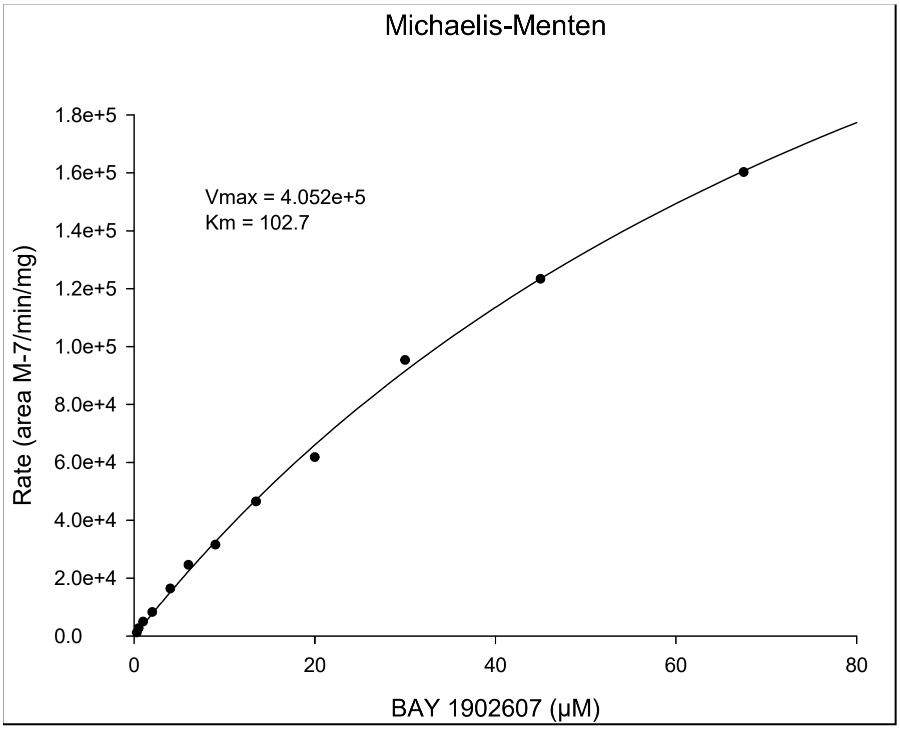

Sum of metabolites M-2, M-4, M-6, M-7, M-8, M-9

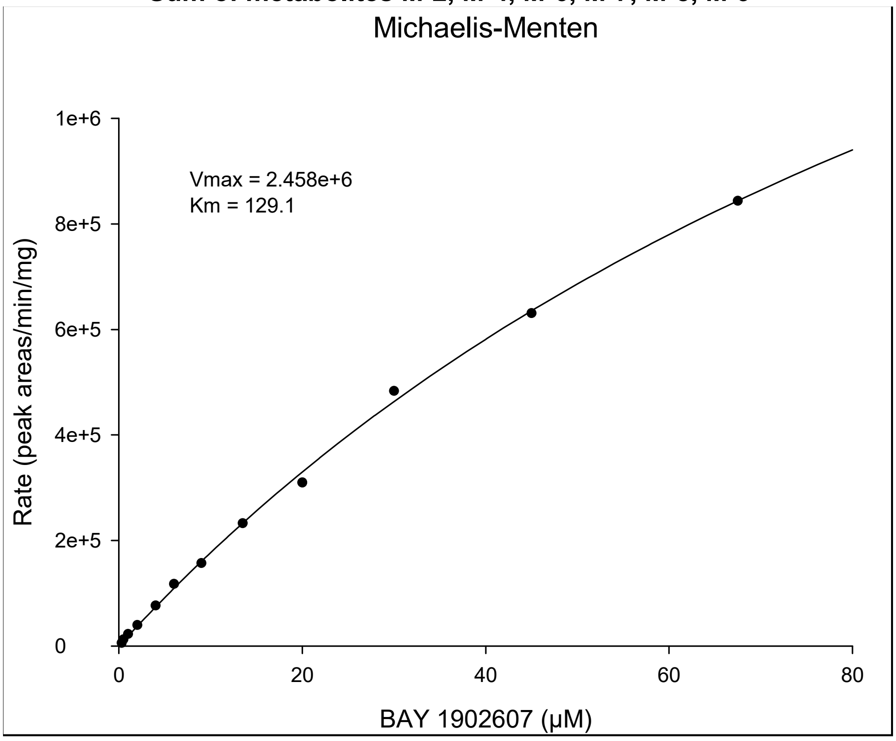

Supplement: Supplementary file 1 [file ijms-26-10177-s001.zip › ijms-3852254-supplementary.pdf]
